# Psychological distress and subjective health status of adolescents living with a sibling with care needs: a population-based study

**DOI:** 10.1265/ehpm.25-00161

**Published:** 2025-11-12

**Authors:** Kohei Enami, Ichiro Kawachi, Tsuguhiko Kato

**Affiliations:** 1 Graduate School of Public Health, St. Luke’s International University, Tokyo, Japan; 2 Department of Social and Behavioral Sciences, Harvard T.H. Chan School of Public Health, Boston, MA, USA

**Keywords:** Sibling, Children/adolescents with care needs, Self-rated health, Psychological distress, Spillover effect, Population study

## Abstract

**Background:**

Growing up with a sibling with a chronic health problem or a disability requiring assistance can affect the lives of the family members in various ways. Previous studies documented health problems among siblings of children with a chronic health problem or a disability. However, these studies are limited in that they tend to rely on small convenience samples of children with specific illnesses/disabilities. This study aims to investigate mental health and self-rated health status of siblings of such children using data from a population study in Japan.

**Methods:**

We used data from the 2016 wave of Japan’s Comprehensive Survey of Living Conditions. The analytic sample included 16,510 adolescents aged 15–19 years who were living with a sibling with or without care needs. The outcomes were psychological distress as defined by K6 score of 13 or higher and poor self-rated health. We examined these health outcomes of adolescents who have a sibling with care needs to relative to adolescents with a sibling without such needs via logistic regression.

**Results:**

Adolescents who live with a sibling with care needs were more likely to have psychological distress (Odds Ratio (OR) 2.47; 95% Confidence Interval (CI), 1.46–4.17) and poor self-rated health (OR 2.21; 95% CI, 1.30–3.75). These associations were more pronounced in post-high school age (18–19 years old) group than in high school age (15–18 years old) group.

**Conclusion:**

The presence of a child with care needs in the household was associated with spillover psychological distress and poorer subjective health among siblings. Providing support for children/adolescents with care needs may have additional benefits in terms of well-being of their siblings.

## Introduction

Attention has been paid to the challenges faced by children/adolescents living with chronic health problems [1]. In the United States, 19.4% of children under 18 had a special health care need in 2019–2020, where children with special health care needs are defined as “those who have or are at increased risk for a chronic physical, developmental, behavioral, or emotional condition and who also require health and related services of a type or amount beyond that required by children generally” [2]. In Japan, the prevalence of special health care needs was 12.5% among 10-year-old children [3]. A related concept is “children in need of medical care,” who continue to require routine medical care including ventilator use and tube feeding after discharge from a neonatal intensive care unit. Children in need of medical care aged 0–19 in Japan doubled between 2005 to 2019 [4]. In Japan, approximately 80% of 15-year-old children were cohabiting with at least one sibling in 2017 [5] and as such, consideration of any potential spillover effects of siblings is important.

However, insufficient attention has been paid from the population health perspective to the potential spillover effects of children with care needs on the health and wellbeing of their siblings. Qualitative evidence indicates that siblings of children in need of medical care experience loneliness, stress, insufficient time with parents, and inability to go out [6]. Previous studies provide evidence that the mental health and behavioral problems of siblings of children with chronic health problems are negatively affected [7–9]. For example, in a Canadian study, siblings of children with a developmental disability experience higher odds of a depression or other mental health diagnosis compared to siblings of children who do not have a developmental disability [10]. Among siblings of children with childhood cancer, those who had been young at the time of diagnosis were at increased risk for hospital contact for mental health problems [11]. A study based on eight high schools in a prefecture in Tokyo metropolitan area in Japan showed that students who had a sibling with chronic illness or disability tended to exhibit poor psychological well-being, anxiety, and behavioral problems [12]. While previous studies generally show an adverse association between having a sibling with a disability and well-being outcomes, this is not universally observed. Middle high school students in Japan with a disabled sibling tended to exhibit lower depressive symptom scores relative to the control group [13]. In a study of rare diseases, siblings reported health-related quality of life comparable to controls [14].

Previous studies primarily relied on relatively small convenience samples of specific disability/disease types [7, 15, 16]. As such, to our knowledge there is no study on this topic based on Japan’s national data set. Adding a population health perspective in our understanding of this topic is crucial for informing public policy regarding the well-being of adolescents with a sibling with chronic conditions. Additionally, previous studies have primarily been conducted in Europe and North America [7, 16]. A broader perspective is important given cultural and institutional variations in how a child’s care needs could affect other family members. Such factors include household structure (the number of children per household and the prevalence of extended families, as well as parents’ preferences for equality among their children), availability of public assistance for care needs (e.g., Japan has established support centers for children in need of medical care and programs to train coordinators for assisting such children), as well as characteristics of the school system including the degree of inclusive education (Japan is regarded as a society with low inclusive schooling [17]).

The purpose of this study is to examine the impact of having a sibling with a disability requiring assistance on adolescents’ self-rated health and mental health in a nationally representative sample of Japan. Given the findings of previous literature that siblings of children with chronic conditions had greater depression rating scale scores [7], we hypothesize that the exposure is adversely associated with mental and general health of adolescents.

## Methods

### Study sample

We used data from the 2016 wave of Comprehensive Survey of Living Conditions, a nationwide repeated cross-sectional survey of households in Japan [18]. Households were sampled through a stratified random sampling method. Of 289,470 households surveyed, 224,641 households responded. Information on adolescents aged 15–19 who were at least high school age at the time of the survey as determined by month of birth was used (n = 25,538).

Adolescents’ and siblings’ care needs status was determined by the response to a survey question regarding whether they required assistance or monitoring due to a disability or a decline in physical functioning. Following the definition of the disability status in a study using the same data set [19], in this study care needs were deemed present if the condition requiring assistance or monitoring had lasted 3 months or longer.

Adolescents who had their own care needs (n = 278), who were married (n = 107), who were not cohabiting with their parents or grandparents (n = 1,738), and who did not have a cohabiting sibling aged 6–29 (n = 5,835) were dropped from the sample. Intuitively, this study aimed to make a comparison between adolescents living with a sibling with care needs and those living with a sibling without care needs, excluding the sibling-pairs not in the same household.

**Fig. 1:**
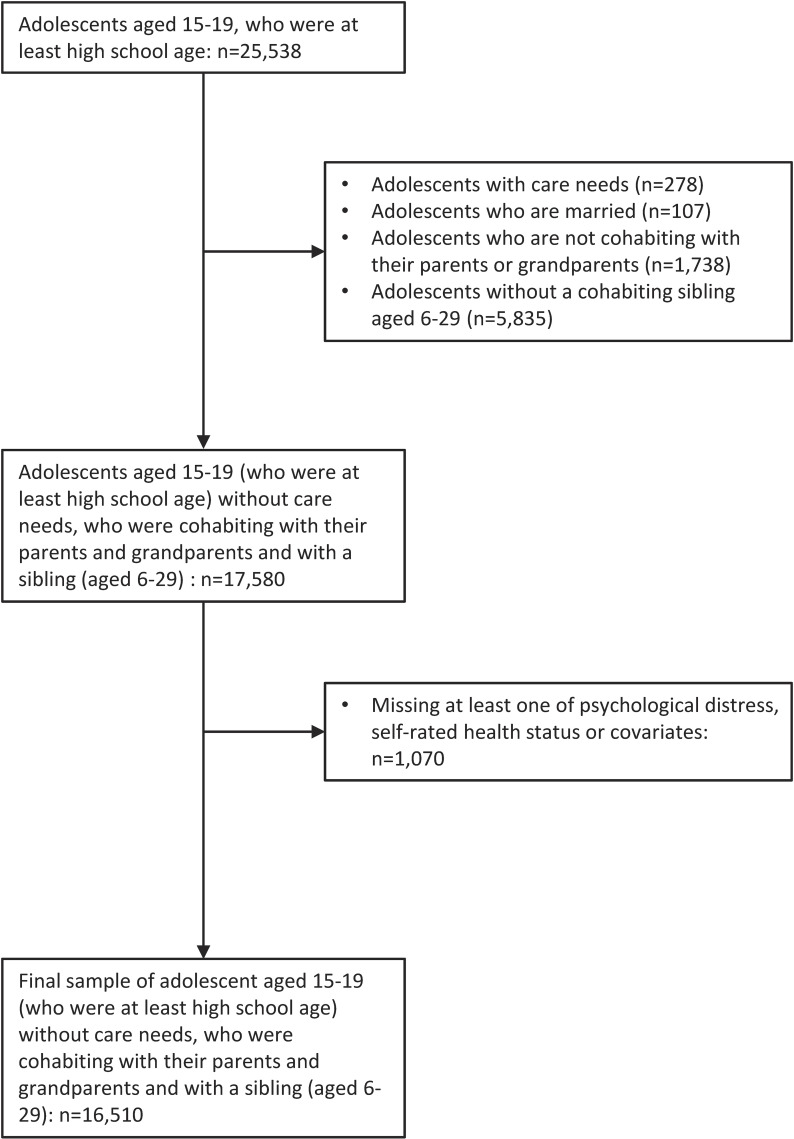
Sample selection process.

Observations were dropped if psychological distress, self-rated health, or one of the covariates was missing (n = 1,070). The final sample consisted of 16,510 adolescents without their own care needs aged 15–19 who were at least high school age, who were cohabiting with their parents and grandparents and with a sibling aged 6–29 (Fig. 1). Ethics approval for this study was obtained at the National Center for Child Health and Development (Approval No: 2023-121).

### Measures

Adolescents’ psychological distress was determined by the Japanese version of Kessler 6 (K6) score [20]. Psychological distress was deemed present if the adolescent exhibited K6 score of 13 or higher, which is indicative of severe mental illness. Adolescents were asked to rate their current health status by choice of poor, fair, good, very good, and excellent. In the analysis self-rated health was dichotomized such that poor and fair health on the 5-point scale were deemed poor self-rated health.

Covariates included age group and sex of the adolescent, whether the adolescent lived in a single parent household or with grandparents, mother’s and father’s educational attainment, number of siblings, and mother’s age at birth of the adolescent. Age group was determined based on the month of birth of the adolescent. The thresholds for high school age group were based on Japan’s academic year which begins in April and ends in March. Specifically, high school age group included those born between April 1998 and March 2001 (who were aged 15–18 at the time of the survey), and post-high school age group included those born between July 1996 and March 1998 (who were aged 18–19 at the time of the survey).

### Statistical analyses

Logistic regression was performed for adolescent psychological distress and poor self-rated health. Subsample analyses included analyses focused on high school age and post-high school age samples, and male and female samples. Subsample analyses were performed for reasons including (i) a previous study in Japan reported more adverse outcomes among male adolescents [12], (ii) perceived responsibilities to provide care for siblings with health problems may vary by the age and gender of the adolescents, and (iii) the social and developmental context of high school age and post-high school age adolescents may vary significantly. Additionally, effect heterogeneity was explored by stratifying the exposure based on sex and age (relative to the focal adolescent) of the sibling with care needs. Standard errors were clustered at household level.

## Results

Table 1 reports the descriptive statistics of the study sample. Psychological distress was more prevalent among adolescents with a sibling with care needs (i.e., exposed adolescents) (7%) than among non-exposed adolescents (3%). Exposed adolescents tended to report poorer self-rated health (28% reporting excellent health and 7% poor or fair health) than nonexposed adolescents (37% excellent health, 3% poor or fair health) on the 5-point scale subjective health measure.

**Table 1:** Descriptive statistics

	**Have a sibling with ** **care needs**			
	**Yes ** **(n = 218)**		**No ** **(n = 16,292)**	
Characteristics of focal adolescent				
Age				
High school age (15–18)	160	73%	11,615	71%
Post-high school age (18–19)	58	27%	4,677	29%
Male	83	38%	8,217	50%
Self-rated health status				
Poor	1	<1%	48	<1%
Fair	14	6%	462	3%
Good	98	45%	6,078	37%
Very good	43	20%	3,642	22%
Excellent	62	28%	6,062	37%
Psychological distress (K6 score 13 or higher)	16	7%	497	3%

Household characteristics				
Single parent household	30	14%	2,109	13%
Household with grandparents	48	22%	3,845	24%
Mother’s educational category				
High school or less	97	45%	7,104	44%
Some college	75	34%	5,072	31%
College	25	11%	1,650	10%
Missing	21	10%	2,466	15%
Father’s educational category				
High school or less	89	41%	6,556	40%
Some college	24	11%	1,712	11%
College or higher	57	26%	4,107	25%
Missing	48	22%	3,917	24%
Number of siblings				
One	109	50%	10,763	66%
Two	81	37%	4,592	28%
Three or more	28	13%	937	6%
Mother’s age at birth of focal adolescent				
<25	34	16%	2,600	16%
25–34	156	72%	11,866	73%
35+	26	12%	1,390	9%
Missing	2	1%	436	3%

Table 2 shows the crude and adjusted association between having a sibling with care needs and psychological distress in the full sample, which includes high school age and post-high school age adolescents. After adjusting for covariates, having a sibling with care needs was associated with an elevated risk of psychological distress (Odds Ratio (OR) 2.47; 95% Confidence Interval (CI), 1.46–4.17). Regarding the notable covariates, male adolescents were less likely to have psychological distress (OR 0.66; 95% CI, 0.55–0.79), and adolescents in single-parent households were more likely to have psychological distress (OR 1.54; 95% CI, 1.00–2.38).

**Table 2:** Association between having a sibling with care needs and mental health problems

	**Full sample**				**Subsample analyses^a^**							
	
	**Crude**		**Adjusted**		**High school ** **group**		**Post-high school group**		**Male adolescent group**		**Female adolescent group**	
					
	**OR**	**95% CI**	**OR**	**95% CI**	**OR**	**95% CI**	**OR**	**95% CI**	**OR**	**95% CI**	**OR**	**95% CI**
Have a sibling with care needs	2.52	1.50–4.23	2.47	1.46–4.17	2.03	1.01–4.08	3.26	1.43–7.42	3.30	1.39–7.82	2.09	1.09–4.03
Age												
High school age (15–18)	Ref.		Ref.						Ref.		Ref.	
Post high school age (18–19)	1.30	1.08–1.56	1.28	1.07–1.54					1.45	1.09–1.94	1.17	0.92–1.50
Male adolescent	0.66	0.55–0.78	0.66	0.55–0.79	0.61	0.49–0.77	0.77	0.57–1.04				
Single parent household	1.53	1.21–1.93	1.54	1.00–2.38	1.28	0.74–2.22	2.29	1.16–4.51	1.06	0.52–2.14	1.99	1.16–3.44
Household with grandparents	1.01	0.82–1.25	1.00	0.80–1.24	0.89	0.68–1.17	1.21	0.85–1.70	1.22	0.89–1.68	0.86	0.64–1.15
Mother’s educational category												
High school or less	Ref.		Ref.		Ref.		Ref.		Ref.		Ref.	
Some college	0.83	0.67–1.03	0.86	0.69–1.07	0.93	0.70–1.23	0.73	0.51–1.06	0.88	0.63–1.25	0.84	0.63–1.13
College or higher	0.94	0.69–1.29	1.04	0.73–1.47	1.12	0.73–1.72	0.90	0.49–1.65	0.96	0.56–1.64	1.11	0.70–1.76
Missing	1.01	0.78–1.32	1.02	0.67–1.56	1.17	0.69–2.00	0.78	0.40–1.51	1.05	0.54–2.06	1.01	0.59–1.74
Father’s educational category												
High school or less	Ref.		Ref.		Ref.		Ref.		Ref.		Ref.	
Some college	1.01	0.73–1.39	1.04	0.75–1.45	0.91	0.61–1.36	1.37	0.81–2.32	1.24	0.77–2.01	0.93	0.60–1.43
College or higher	0.90	0.71–1.14	0.91	0.69–1.19	0.78	0.56–1.09	1.22	0.78–1.90	1.25	0.83–1.86	0.72	0.50–1.04
Missing	1.29	1.03–1.61	1.02	0.66–1.56	1.04	0.61–1.79	0.89	0.46–1.74	1.36	0.69–2.69	0.82	0.47–1.42
Number of siblings												
One	Ref.		Ref.		Ref.		Ref.		Ref.		Ref.	
Two	0.87	0.71–1.07	0.88	0.71–1.08	0.80	0.62–1.04	1.04	0.74–1.46	0.89	0.65–1.23	0.86	0.65–1.12
Three or more	0.72	0.44–1.18	0.71	0.43–1.16	0.74	0.45–1.24	0.64	0.25–1.64	0.74	0.36–1.54	0.68	0.39–1.20
Mother’s age at birth of focal adolescent												
<25	Ref.		Ref.		Ref.		Ref.		Ref.		Ref.	
25–34	0.95	0.75–1.22	1.01	0.78–1.29	0.92	0.68–1.25	1.21	0.80–1.83	0.88	0.60–1.29	1.10	0.80–1.52
35+	0.96	0.66–1.39	1.02	0.70–1.49	0.99	0.63–1.55	1.09	0.54–2.24	0.81	0.44–1.48	1.20	0.74–1.94
Missing	0.92	0.51–1.66	0.62	0.25–1.59	0.70	0.21–2.37	0.56	0.14–2.24	0.63	0.15–2.66	0.62	0.18–2.13

N		16,510		16,510		11,775		4,735		8,300		8,210

^a^Adjusted odds ratios are reported for subsample analyses.

Table 2 also shows the results of stratified analyses: adolescents in the high school age; adolescents in the post-high school age; adolescents who are male; and adolescents who are female. The association between having a sibling with care needs and psychological distress appeared to be larger in post-high school age group (OR 3.26; 95% CI, 1.43–7.42) than in high school age group (OR 2.03; 95% CI, 1.01–4.08), and larger in male adolescents (OR 3.30; 95% CI, 1.39–7.98) than in female adolescents (OR 2.09; 95% CI, 1.09–4.03).

Table 3 shows the results of logistic regressions for the outcome of self-rated health with full sample unadjusted and adjusted models, followed by results using subsamples where adjusted odds ratios are reported. Adjusted full sample analysis indicates that having a sibling with care needs was associated with poor subjective health (OR 2.21; 95% CI, 1.30–3.75). The association appears to be stronger in the post-high school sample (OR 3.59; 95% CI, 1.59–8.08) than in the high school sample (OR 1.62; 95% CI, 0.78–3.35). The association in female adolescent sample was statistically significant at 5% level (OR 2.38; 95% CI, 1.28–4.44), but not in the male adolescent sample (OR 1.85; 95% CI, 0.67–5.11).

**Table 3:** Association between having a sibling with care needs and self-rated health

	**Full sample**				**Subsample analyses^a^**							
	
	**Crude**		**Adjusted**		**High school group**		**Post-high school group**		**Male adolescent group**		**Female adolescent group**	
					
	**OR**	**95% CI**	**OR**	**95% CI**	**OR**	**95% CI**	**OR**	**95% CI**	**OR**	**95% CI**	**OR**	**95% CI**
Have a sibling with care needs	2.28	1.34–3.89	2.21	1.30–3.75	1.62	0.78–3.35	3.59	1.59–8.08	1.85	0.67–5.11	2.38	1.28–4.44
Age												
High school age (15–18)	Ref.		Ref.						Ref.		Ref.	
Post high school age (18–19)	1.07	0.89–1.30	1.08	0.89–1.30					0.94	0.69–1.26	1.19	0.93–1.53
Male adolescent	0.75	0.63–0.90	0.76	0.64–0.91	0.82	0.66–1.00	0.64	0.46–0.89				
Single parent household	1.14	0.89–1.46	1.17	0.76–1.78	1.43	0.83–2.47	0.79	0.40–1.56	1.05	0.56–1.98	1.27	0.71–2.26
Household with grandparents	0.94	0.76–1.15	0.95	0.76–1.18	0.92	0.71–1.19	1.02	0.70–1.49	0.90	0.65–1.24	0.99	0.75–1.31
Mother’s educational category												
High school or less	Ref.		Ref.		Ref.		Ref.		Ref.		Ref.	
Some college	0.93	0.76–1.14	0.98	0.79–1.23	1.00	0.77–1.30	0.94	0.63–1.41	1.01	0.73–1.41	0.96	0.71–1.30
College or higher	1.21	0.92–1.60	1.35	0.97–1.88	1.67	1.16–2.41	0.68	0.33–1.41	1.51	0.95–2.39	1.22	0.78–1.92
Missing	0.72	0.54–0.96	0.71	0.46–1.11	0.65	0.37–1.17	0.80	0.41–1.56	0.60	0.30–1.18	0.82	0.45–1.47
Father’s educational category												
High school or less	Ref.		Ref.		Ref.		Ref.		Ref.		Ref.	
Some college	0.72	0.52–1.01	0.72	0.51–1.02	0.61	0.40–0.93	1.10	0.60–2.00	0.88	0.53–1.45	0.60	0.37–0.97
College or higher	0.91	0.73–1.13	0.82	0.63–1.07	0.68	0.50–0.92	1.35	0.84–2.17	0.83	0.57–1.22	0.82	0.58–1.17
Missing	0.95	0.76–1.19	1.00	0.66–1.5	0.78	0.46–1.33	1.68	0.88–3.2	1.16	0.64–2.10	0.88	0.50–1.55
Number of siblings												
One	Ref.		Ref.		Ref.		Ref.		Ref.		Ref.	
Two	0.94	0.77–1.15	0.94	0.77–1.15	0.81	0.63–1.03	1.32	0.94–1.86	1.04	0.78–1.39	0.87	0.66–1.14
Three or more	0.82	0.55–1.23	0.80	0.52–1.21	0.81	0.49–1.32	0.79	0.34–1.82	0.69	0.36–1.33	0.89	0.53–1.51
Mother’s age at birth of focal adolescent												
<25	Ref.		Ref.		Ref.		Ref.		Ref.		Ref.	
25–34	1.01	0.87–1.03	1.02	0.79–1.30	0.93	0.70–1.25	1.25	0.80–1.96	0.87	0.61–1.24	1.15	0.82–1.62
35+	1.02	0.92–1.20	1.01	0.69–1.47	0.87	0.56–1.36	1.50	0.75–3.01	0.87	0.50–1.52	1.14	0.69–1.89
Missing	0.64	0.67–1.03	0.79	0.32–1.93	0.76	0.23–2.49	0.97	0.25–3.8	0.66	0.16–2.7	0.88	0.26–2.91

N		16,510		16,510		11,775		4,735		8,300		8,210

The dependent variable is poor self-rated health, defined as having one of the lowest two health states (poor or fair health) on the five-category self-rated health scale.

^a^Adjusted odds ratios are reported for subsample analyses.

Finally, Table 4 shows the analysis on the potential heterogeneity of associations by age and sex of the sibling with care needs. The point estimates of the odds ratios for having a younger sibling with care needs are 3.23 and 1.63 for psychological distress and self-rated health, respectively. For older siblings these point estimates were 1.70 and 3.14. Regarding the sex of sibling with care needs, the point estimates of the odds ratios for having a female sibling with care needs 2.90 and 1.82 for psychological distress and self-rated health, respectively, and for a male sibling with care needs, the odds ratios were 2.26 and 2.39. The confidence intervals for these results were wide, and there was no clear pattern regarding the heterogeneity of the associations by age and sex of the sibling with care needs.

**Table 4:** Heterogeneity of associations by characteristics of the sibling with care needs

**Dependent variable:**	**Psychological distress**		**Self-rated health^a^**	
**Model:**	**Logistic regression**		**Logistic regression**	
	**OR**	**95%CI**	**OR**	**95%CI**
A. Exposure stratified by relative age^b^				
Do not have a sibling with care needs	Ref.		Ref.	
Sibling with care needs is older than focal adolescent	1.70	0.68–4.27	3.14	1.58–6.26
Sibling with care needs is younger than focal adolescent	3.23	1.71–6.09	1.63	0.53–3.25

B. Exposure stratified by sex of sibling with care needs^c^				
Do not have a sibling with care needs	Ref.		Ref.	
Sibling with care needs is male	2.26	1.17–4.39	2.39	1.29–4.44
Sibling with care needs is female	2.90	1.26–6.72	1.82	0.67–4.95

Regressions control for the same set of covariates as the models reported in Tables 2 and 3.

^a^The dependent variable is poor self-rated health, defined as having one of the lowest two health states (poor or fair health) on the five-category self-rated health scale.

^b^N = 16,505 for psychological distress (five observations dropped due to collinearity). N = 16,510 for self-rated health.

^c^N = 16,510.

## Discussion

This study found adolescents living with a sibling with care needs are more likely to suffer from psychological distress and poorer general health status relative to adolescents living with a sibling without care needs using a large population-based data in Japan. To our knowledge this is the first study in Japan based on a nationally representative data set to study the spillover effect of having a sibling with care needs on adolescents mental and general health.

Another novelty of this study is that we focused on having a sibling with care needs as an exposure, which is different from having a sibling with special health care needs [3] or having care responsibilities.

The findings are generally consistent with previous studies using clinical and population-based samples of in that having a sibling with chronic conditions is generally associated with poorer mental health [7]. Similarly, findings are consistent with previous studies that used presence of siblings with non-specific long-term health conditions or disabilities as the exposure [12, 15]. While previous research centered on the outcomes at younger ages, this study showed the associations in adolescents aged 15–19 – and the associations appeared to be more pronounced in post-high school age (18–19) group than in high school age (15–18) group.

This study also adds to our understanding regarding the extent of the spillover effects arising from family members’ health problems. As to the impact on parents, children’s disability status is associated with poorer mental health of their mothers [21] and fathers [19]. Children’s special care needs status was also associated with mothers’ anxiety and depression [3]. This study confirms the impacts on siblings using population-based data.

Another important background of this study has been the growing attention being paid to well-being of young carers. The role as a young carer is shown to be associated with poorer mental health among adolescents [22]. Study of young carers is related but separate from this study in that (i) this study aimed to capture the overall impact of having a sibling with care needs regardless of the mechanisms, while the young carer research focuses on impact arising from the responsibility to provide care for their family members with chronic conditions, and (ii) in young carer literature, family members requiring care are not limited to siblings, but include grandparents or parents.

### Potential mechanisms

There are several potential mechanisms through which the health and well-being of healthy adolescents (i.e., free of chronic conditions) may be affected by the presence of a sibling with care needs. First, children’s health problems may negatively affect parental mental health [19, 21], which may in turn spill over to affect the psychological well-being of siblings, potentially via worsened quality of parenting [23]. Second, parents may allocate more resources to the care of the child with a chronic condition, leaving less resources for healthy siblings. Theoretically, parents may respond to the health needs of children by either reinforcement (investing more in healthier children) or compensation (investing more in unhealthy children in an attempt to reduce the inequality in terms of health and well-being among children), while the empirical literature indicates no clear direction of parental response (see [24]). Third, a child’s disability may put financial pressures on the family through direct out-of-pocket expenditures associated with the disability, and through impacts on parents’ capacity to engage in paid work [25, 26]. Fourth, healthy siblings may be recruited to provide care of a sibling with a disability. While this study did not focus on caregiving roles of adolescents, poorer health of young carers compared to non-caregiving peers has been documented in the literature [22, 27].

### Limitations

There are several limitations. First, the measurement of care needs was based on the response by one of the household members and was not based on specific diagnostic criteria. Also, we did not explicitly assess the intensity of care needs or the presence of external assistance. Second, the association between adolescent outcomes and having a sibling with care needs may be due to a common cause shared by the focal adolescent and the sibling (e.g., genetics), rather than being a causal relationship. Third, given the observational nature of the study with a limited set of covariates, caution is warranted regarding the causal interpretation. In particular, there could be residual confounding by unaccounted household characteristics such as family income, social support, and access to services. Fourth, we did not investigate the specific mechanisms linking the exposure and the outcomes. Fifth, because some adolescents leave their home after high school, the post-high school adolescents in the current sample who were cohabiting with their family were a subset of the adolescents of this age group. This nonrandom selection may have led to biases. For example, if those with a sibling with greater care needs may have disproportionately opted to stay with the family after high school, the associations between exposure and outcomes may have been overestimated. Lastly, this study looked at self-rated health and psychological distress of adolescents, other dimensions of well-being, including educational outcomes, employment, and family formation [28–30], as well as the experiences in adulthood [31], may be impacted by having a sibling with a disability.

## Conclusion

This study showed that the adolescents living with a sibling with care needs were more likely to suffer from psychological distress and self-rated overall health status relative to adolescents living with a healthy sibling using Japan’s population-based survey.

Public policy implications of this study are three-fold. First, the findings on poorer mental and general health of adolescents living with a sibling with care needs underscore the importance of raising the parental awareness as well as public and schools’ understanding of sibling issues to better address the needs of these adolescents [32, 33]. Second, the findings point to the need to identify and implement effective interventions for improving the well-being of such adolescents. These interventions may include sibling support groups (e.g., involving components such as discussion, problem solving, and coping) and parent-focused interventions, but results of studies evaluating these interventions are varied [34–36]. Third, providing support for children/adolescents with care needs may have collateral benefits in terms of supporting the well-being of their siblings. Quantifying these benefits may provide justification for additional resources for the treatment of chronic health problems among children/youth.

Future research should address the specific mechanisms underlying these associations and differential implications of different disease types, as well as potential protective factors (e.g., emotional support) for affected adolescents [37, 38]. Evidentiary basis for interventions such as group sessions for siblings to support the well-being of affected adolescents should be strengthened, given the limited evidence due to methodological issues including sample size, study design (e.g., pre-post design), and short follow-up period [34, 35]. Future research should also address long-term consequences of such exposure such as long-term physical and mental health, health services use, educational attainment, labor market outcomes, and family formation.

## Declarations

### Availability of data and material

Data cannot be shared because use of data from Comprehensive Survey of Living Conditions requires permission by the Ministry of Health, Labour and Welfare.
